# In Vitro Growth Conditions Boost Plant Lipid Remodelling and Influence Their Composition

**DOI:** 10.3390/cells10092326

**Published:** 2021-09-06

**Authors:** Sylwia Klińska, Sara Kędzierska, Katarzyna Jasieniecka-Gazarkiewicz, Antoni Banaś

**Affiliations:** Intercollegiate Faculty of Biotechnology, University of Gdansk and Medical University of Gdansk, 80-307 Gdansk, Poland; s.kedzierska.439@studms.ug.edu.pl (S.K.); katarzyna.jasieniecka@biotech.ug.edu.pl (K.J.-G.); antoni.banas@biotech.ug.edu.pl (A.B.)

**Keywords:** lipid remodelling, phosphatidylcholine, LPCAT, LPEAT, *in vitro* and *in vivo* growth conditions, acyl-lipid metabolism

## Abstract

Acyl-lipids are vital components for all life functions of plants. They are widely studied using often *in vitro* conditions to determine inter alia the impact of genetic modifications and the description of biochemical and physiological functions of enzymes responsible for acyl-lipid metabolism. What is currently lacking is knowledge of if these results also hold in real environments—in *in vivo* conditions. Our study focused on the comparative analysis of both *in vitro* and *in vivo* growth conditions and their impact on the acyl-lipid metabolism of *Camelina sativa* leaves. The results indicate that *in vitro* conditions significantly decreased the lipid contents and influenced their composition. In *in vitro* conditions, galactolipid and trienoic acid (16:3 and 18:3) contents significantly declined, indicating the impairment of the prokaryotic pathway. Discrepancies also exist in the case of acyl-CoA:lysophospholipid acyltransferases (LPLATs). Their activity increased about 2–7 times in *in vitro* conditions compared to *in vivo*. *In vitro* conditions also substantially changed LPLATs’ preferences towards acyl-CoA. Additionally, the acyl editing process was three times more efficient in *in vitro* leaves. The provided evidence suggests that the results of acyl-lipid research from *in vitro* conditions may not completely reflect and be directly applicable in real growth environments.

## 1. Introduction

Plant cell membranes contain a double layer structure, built mainly by glycerolipids and proteins. The composition of these lipids substantially influences the fluidity and permeability of the membrane. In plants, two groups of glycerolipids can be distinguished: glycolipids and phospholipids. The former is characterised by the presence of a sugar group attached to the *sn*-3 position of the glycerol backbone, whereas in the latter the phosphate group occupies this position. Galactolipids dominate the glycerolipid pool and constitute up to 85% of all plant membrane lipids [1]. The main representatives of this group are MGDG (monogalactosyldiacylglycerol) and DGDG (digalactosyldiacylglycerol). They are present mostly in the thylakoid membrane of the chloroplast, where they form a matrix indispensable for proper photochemical reactions and the transport of electrons during photosynthesis [2,3,4]. The second group of compounds—phospholipids—consists mainly of phosphatidylcholine (PC), phosphatidylethanolamine (PE) and to a lesser extent phosphatidic acid (PA), phosphatidylserine (PS) and phosphatidylinositol (PI). Phospholipids occur in all plant cell membranes; however, their composition may differ between the cellular compartments. Phosphatidylcholine is the dominant phospholipid, which is also a key substrate for the production of polyunsaturated fatty acids and some uncommon fatty acids [5]. The second most abundant phospholipid is phosphatidylethanolamine. Its contents and composition strongly influence plant cell physiology by regulating the fluidity and conformation of the membranes, and by affecting the autophagy process [6,7,8]. The third mentioned phospholipid—phosphatidic acid—is present in limited quantities, as most of it is used (right after its synthesis) for membrane and storage lipid biosynthesis [5]. Moreover, PA may play an essential role in signal transduction during stress conditions, when its elevated amount is produced via phospholipase D activity [9]. Similarly, PS and PI are present in the membrane in limited amounts, and besides their role in the formation of membrane structures, they can participate in the cell signalling process [10,11].

The biosynthesis of both groups of compounds is connected with diacylglycerol (DAG) pool production. DAG may be synthesised via two pathways: eucaryotic (mainly leading to phospholipid production) or procaryotic (mainly responsible for galactolipid formation). Each one is distinguished by another place of occurrence and substrate specificity of the enzymes involved [5,12]. The initial step is the same for both pathways—a reaction catalysed by glycerol-3-phosphate acyltransferase (GPAT) takes place and performs the acylation of the *sn*-1 position of the glycerol backbone. Donors of fatty acids for this reaction are acyl-ACPs (for reactions occurring in the plastid) and acyl-CoA molecules (for reactions occurring in the cytosol). The same kind of substrates are also used by the next enzymes to form these pathways—acyl-CoA:lysophosphoshatidic acid acyltransferase (LPAAT)—producing phosphatidic acid. Both enzymes exhibit different substrate specificities depending on their localisation. In the reactions catalysed by GPAT in the plastids, oleic acid is mainly attached to the *sn*-1 position, whereas in ER it is palmitic acid [11,13]. The *sn*-2 position of the formation in the plastid lysophosphatidic acid is exclusively esterified by palmitic acids and in ER mainly by oleic acid. The last step of the *de novo* formation of DAG is the dephosphorylation step conducted by phosphatidate phosphatase (PAP). The pool of *de novo*-formed DAG is further supplemented by the DAG molecules created, e.g., via phospholipase C activity or the action of CDP-choline:diacylglycerol cholinephosphotransferase (CPT) or phosphatidylcholine:diacylglycerol cholinephosphotransferase (PDCT). The DAG molecules created in the cytosol can be transferred to the plastid and used for chloroplast lipid production containing unsaturated 18C fatty acids in the *sn*-2 position, while DAG formed in the plastid can be transferred to the cytosol especially in “16:3 plants” [5,14,15,16]. Both in plastids and in the cytosol, further formation of phospholipids or galactolipids requires the activation of one of the substrates: DAG or polar headgroup. The biosynthesis of, e.g., PE and PC requires CDP-choline or CDP-ethanolamine, respectively, and the biosynthesis of MGDG requires UDP-galactose. On the contrary, the biosynthesis of, e.g., PS and PI needs CDP-DAG [15,16,17].

After the *de novo* formation of phospholipids, they undergo constant remodelling; the deacylation process occurs and lysophospholipids are formed. In turn, these lysophospholipids are reacylated and new species of phospholipids with another set of fatty acids can be formed. Acyl-CoA:lysophospholipid acyltransferases (LPLATs) play the key role in such acyl editing of phospholipids. These enzymes are widespread among organisms and are responsible for phospholipid production from lysophospholipids (LPL) and acyl-CoA. Different groups of LPLATs can be distinguished based on their acyl acceptor preference. For instance, acyl-CoA: lysophosphatidylcholine acyltransferases (LPCATs) are characterised by the highest specificity toward lysophosphatidylcholine, acyl-CoA:lysophosphatidylethanolamine acyltransferase (LPEATs) toward lysophosphatidylethanolamine and previously mentioned LPAATs toward lysophospatidic acid. The deacylation process can be carried out by phospholipases, enzymes of the PDAT type (phospholipid:diacylglycerol acyltransferases) and LPLATs via backward reaction [18,19,20,21,22]. The relative role of the mentioned reactions in the formation of lysophospholipids is so far poorly studied; however, it may differ depending on remodelled phospholipids, plant organs and physiological stage [21,22].

From among LPLAT enzymes, LPCATs seem to play the main role in the acyl editing process. However, LPEATs and LPAATs also revealed such potential, at least in *Camelina sativa* seeds [21,22]. Due to the dual activity of LPLATs, they may play an essential role not only in the remodelling process of phospholipids, but also in adjusting the cytoplasmic acyl-CoA pool by suppling it with acyl-CoAs containing fatty acids derived, e.g., from remodelled PC. The effect of the environment on phospholipid remodelling and LPLAT activity is not well characterised. So far, only the role of LPEATs has been investigated. It was shown by Klińska et al. [23] that the activity and substrate specificity of LPEAT enzymes present in leaves are strongly regulated by temperature, which makes them a sensor of external thermal changes. Information concerning the remodelling of galactolipids is practically unavailable.

In our study, we compared two plant growth conditions: *in vivo* (soil pots; growth chamber) and *in vitro* (agar plates; liquid culture). The *in vivo* method imitates conditions closest to the environmental ones. As regards *in vitro* cultivation, this is currently the most commonly used technique for, e.g., the production of new plant varieties or secondary metabolites. In addition, this type of breeding is widely used in scientific research in the field of biology, biotechnology or agriculture in order to deepen the basic knowledge of plant physiology. It has a significant advantage over *in vivo* culture due to the possibility of controlling the breeding conditions, the possibility of creating the most favourable conditions for plant development or the possibility to breed without biotic stress [24]. On the other hand, the creation of artificial conditions can alter the activity of many biological processes in the cell, which may not be replicated in standard *in vivo* conditions. Studies comparing the types of cultures mainly concentrate on their influence on the production of secondary metabolites. Some compounds can be produced *in vitro* with greater intensity or the same as *in vivo*, or may even not be produced at all [25,26]. Moreover, the extracted substances derived from different plant cultivation conditions may show different properties, e.g., antioxidant or antibacterial [27].

Recently, many studies in the field of lipid biochemistry have also been conducted based on the use of *in vitro* cultures. Most of them concern the determination of the activity of enzymes related to lipid biosynthesis, mainly focusing on the comparison of wild lines and genetically modified ones or testing the impact of diverse abiotic stresses [28,29,30,31]. These studies often do not take into account the effect of the introduced modifications on plants grown under standard conditions (*in vivo*), which might be completely different, such as in the case of secondary metabolite production. The lack of knowledge about the impact of plant breeding methods on acyl-lipid metabolism, especially in vegetative tissue, drove us to conduct the first investigation into this issue. In the presented studies, we investigate the composition of acyl-lipids in leaves from plants cultivated in *in vivo* and *in vitro* growth conditions. We also determine the activity and substrate preference of different groups of acylo-CoA:lysophospholipid acyltransferases, the enzymes related to acyl-lipid biosynthesis and especially to the phospholipid remodelling process. Additionally, we evaluate the intensity of the phospholipid remodelling process occurring during *in vivo* and *in vitro* growth conditions.

## 2. Materials and Methods

### 2.1. Plant Material and Growth Conditions

Plant material was derived from *Camelina sativa* L. Crantz, cv. Suneson growing at two different conditions: *in vivo* and *in vitro*. For *in vivo* conditions, seeds of *C. sativa* were planted in soil and cultivated in a growth chamber at 23 °C with relative humidity at about 60% and photoperiod set for 16 h of light (120 µmol photons m^−2^s^−1^) and 8 h of darkness. After approximately 35 days from sowing, before plants started to bloom, leaves were harvested for further analysis. In case of leaf material derived from *in vitro* conditions, firstly *C. sativa* seeds were planted on plates containing: 2% sucrose, 0.8% agar and 0.5 × Murashige and Skoog (MS) medium, preceded by surface sterilisation of seeds. Subsequently, after 10 days, well-developed seedlings were transferred into the liquid culture containing 0.5 × MS medium supplemented with 2% sucrose for the next 14 days. *In vitro* cultivation was conducted in long-day photoperiod at 23 °C with shaking (100 rpm).

### 2.2. Lipid Analysis

Lipid extraction from *in vivo* and *in vitro* leaves or from microsomal fractions prepared from these leaves was conducted according to the modified method described by Bligh and Dyer [32]. The tissues/microsomes were homogenised in 3.75 mL of chloroform:methanol (1:2, *v*/*v*) followed by the addition of 1.25 mL of 0.15 M acetic acid, 1.25 mL of chloroform and 1.25 mL of water. The chloroform fractions, containing lipids, were collected and separated by TLC on Silica gel 60 plates (Merck, Darmstadt, Germany) with chloroform:methanol:acetic acid:water (90:15:10:2.5, *v*/*v*/*v*/*v*) as solvent system. The plates with separated lipid classes were sprayed with 0.05% primuline solution and visualised under UV light. Based on used lipid standard, areas of gel containing appropriate lipid classes were scraped off and methylated in situ on the gel with 2% H_2_SO_4_ in dry methanol (45 min at 90 °C). The fatty acid methyl esters were extracted by addition of 3 mL of hexane and 2 mL of water. The internal standard—methyl heptadecanoate (17:0-Me)—was added right after methylation. Analysis of contents and composition of fatty acid methyl esters of the prepared samples was conducted by gas–liquid chromatography (Shimadzu; GC-2010) equipped with a fame ionisation detector (FID) and a 60 m × 0.25 mm CP-WAX 58-CB fused-silica column (Agilent Technologies, Santa Clara, CA, USA).

### 2.3. Microsomal Membrane Preparation

Isolation of membrane fraction from *in vivo* and *in vitro* leaves was performed according to the method previously described by Klińska et al. [21]. In summary, the collected leaf material was thoroughly ground in glass homogenisers with extraction buffer (0.1 M potassium phosphate buffer—pH 7.2, 1 mg/mL of BSA, 0.33 M sucrose and catalase (1000 U/mL)). Obtained homogenates were filtered through Miracloth and centrifuged at 20,000× *g* for 12 min to get rid of non-ground tissues and undesirable cell compartments. Supernatants were centrifuged for the second time at 100,000× *g* for 90 min and the resulting pellets—containing microsomal fraction—were resuspended in 0.1 M potassium phosphate buffer (pH 7.2). Aliquots of microsomal fraction were collected for measurement of their “concentration” via determination of phosphatidylcholine contents by method described above. Microsomal fractions were stored at −80 °C for further analysis.

### 2.4. Enzyme Assay

Enzyme assay determining the activity and the substrate specificity of the three acyl-CoA:lysophospholipid acyltransferases—LPAAT, LPCAT and LPEAT—was conducted on microsomal fractions derived from *in vivo* and *in vitro C. sativa* leaves. For enzymatic reactions, previously established parameters for these enzymes present in seeds and leaves were used [21,23]. As acyl donors, ten various acyl-CoAs were used: decanoyl-CoA ([^14^C]10:0-CoA), lauroyl-CoA ([^14^C]12:0-CoA), myristoyl-CoA ([^14^C]14:0-CoA), palmitoyl-CoA ([^14^C]16:0-CoA), stearoyl-CoA ([^14^C]18:0-CoA), oleoyl-CoA ([^14^C]18:1-CoA), linoleoyl-CoA ([^14^C]18:2-CoA), linolenoyl-CoA ([^14^C]18:3-CoA), eicosenoyl-CoA ([^14^C]20:1-CoA) and erucoyl-CoA ([^14^C]22:1-CoA). Mentioned [1-^14^C]acyl-CoAs were synthesised according to the modified method described by Sánchez et al. [33] by using appropriate [1-^14^C]fatty acids (purchased from Larodan AB, Sweden or American Radiolabeled Chemicals, MO, USA) and coenzyme A (Sigma-Aldrich, MO, USA).

For determination of the activity of LPCAT, LPEAT and LPAAT type of enzymes in the prepared microsomal fractions, to the reaction mixtures we added, respectively: 5 nmol of exogenous *sn*-1-18:1-lysophosphatidylcholine, 5 nmol *sn*-1-18:1-lysophosphatidylethanolamine or 5 nmol of *sn*-1-18:1-lysophoshatidic acid together with 5 nmol of appropriate [^14^C]acyl-CoA and aliquots of microsomal fractions (equivalent to 0.2 nmol and 0.5 nmol of the endogenous PC). Reaction mixtures were filled up to 100 µL with 40 mM potassium buffer (pH 7.2). Reactions were carried out at 30 °C for 30 min (for LPCAT) and for 60 min (for LPAAT and LPEAT) with continuous shaking (1250 rpm). Enzymatic reactions were terminated by addition of 375 μL of chloroform:methanol (1:2; *v*:*v*), 5 µL of glacial acetic acid and 125 μL of chloroform. Following mixing and centrifugation, chloroform fractions were collected and separated by thin-layer chromatography on silica gel 60 plates (Merck, Darmstadt, Germany) using polar solvent system (chloroform:methanol:acetic acid:water; 90:15:10:2,5; *v*:*v*:*v*:*v*). The reaction products, [^14^C]-PC, [^14^C]-PE or of [^14^C]-PA were visualised and quantified using electronic autoradiography (Instant Imager, Packard Instrument Co., Meriden, CT, USA).

For measurement of the intensity of remodelling of PC, PE and PA and the effect of different acyl-CoAs on this process, to the reaction mixture aliquots of microsomal fractions containing 10 nmol of endogenous PC together with 10 nmol of oleoyl-CoA ([^14^C]18:1-CoA), linoleoyl-CoA ([^14^C]18:2-CoA) or linolenoyl-CoA ([^14^C]18:3-CoA) and 1 mg of BSA were added. The reaction mixtures were filled up to 100 µL with 40 mM potassium buffer (pH 7.2) and incubated at 30 °C with continuous shaking (1250 rpm) for 5 and 60 min (modified method described by Klińska et al. [23]). Reactions were stopped as described above for LPLAT activity assays. The reaction products ([^14^C]PC, [^14^C]PE and [^14^C]PA) were analysed as described above.

## 3. Results

### 3.1. Contents and Composition of Fatty Acids of Acyl-Lipids in Analysed Leaves

To estimate the acyl-lipid contents in the analysed tissues, the aliquots of chloroform extracts were evaporated to dryness and underwent a methylation procedure (see Materials and Methods). The obtained fatty acid methyl esters were then analysed on GC. The peaks of separated fatty acids were identified by comparison of their retention times with the retention time of fatty acid standards. The concentration of a given fatty acid in the analysed samples was obtained by comparison of the size of its peak with the size of the peak of the internal standard (17:0-Me). Both calculations were performed automatically by software connected to GC.

**Figure 1:**
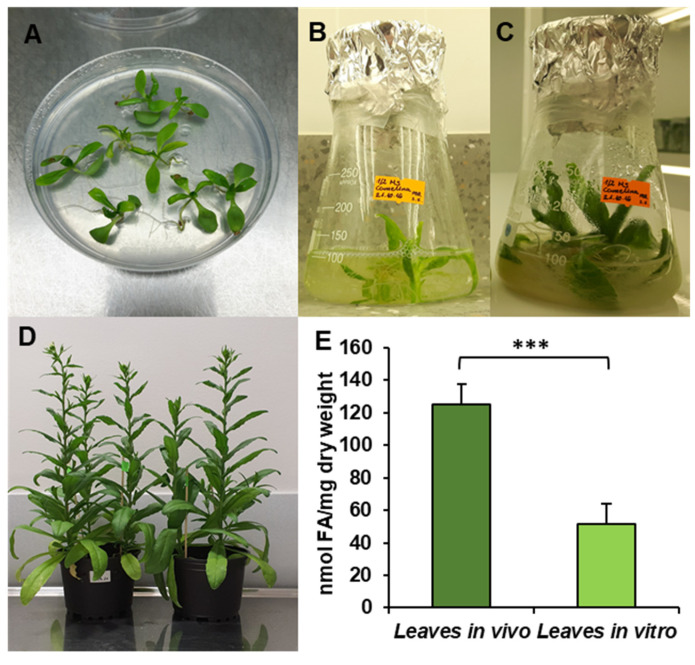
Cultivation conditions—*in vitro* (**A**–**C**), *in vivo* (**D**) and fatty acid contents in *C. sativa* tested leaves (**E**). (**A**) Ten-day-old seedling before transfer to liquid culture; (**B**) 17-day-old seedling, week after transfer to liquid conditions; (**C**) 24-day-old seedling used for microsomal preparation and lipid analysis; (**D**) *in vivo* plants used for the experiments; (**E**) contents of fatty acids in leaves cultivated *in vivo* and *in vitro*. Mean values and SD are presented (data from at least three independent assays). Asterisks indicate significant difference between fatty acid contents in leaves cultivated *in vivo* and *in vitro* in a two-tailed Student’s *t*-test: *** *p* ≤ 0.001.

**Figure 2:**
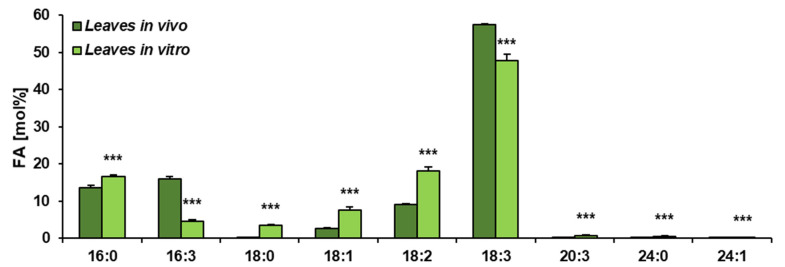
Fatty acid composition of acyl-lipids present in *C. sativa* leaves cultured in *in vivo* and *in vitro* conditions. Mean values and SD are presented (data from at least three independent assays). Asterisks indicate significant difference between relative amounts of each fatty acid of acyl-lipids in leaves cultivated *in vivo* and *in vitro* in a two-tailed Student’s *t*-test: *** *p* ≤ 0.001.

The obtained results showed that the total acyl-lipid contents in the analysed *in vivo* and *in vitro* leaves of *C. sativa* differed significantly. *In vivo* leaves contained over 2.5 times more acyl-lipids per unit of dry weight than their counterparts from *in vitro* cultures (Figure 1). The dominating fatty acid in acyl-lipids of *in vivo* leaves was linolenic acid (18:3) followed by hexadecatrienoic acid (16:3), palmitic acid (16:0), linoleic acid (18:2) and oleic acid (18:1). Their relative amounts accounted for around: 55%, 17%, 13%, 8% and 3%, respectively. The other identified fatty acids (18:0, 20:3, 20:4 and 24:1) did not exceed 1% (individually) of the total fatty acid contents. In acyl-lipids of *in vitro* leaves, 18:3 accounted for about 48% of the total fatty acids, 18:2 for about 18%, 16:0 for 17%, 18:1 for 6%, 16:3 for 4% and 18:0 for about 3%. The other detected fatty acids (20:3, 20:4 and 24:1) constituted a very minor part of the total fatty acids, similarly to *in vivo* leaves (Figure 2). Thus, the fatty acid composition of acyl-lipids of *in vitro* leaves differed substantially from that of *in vivo* leaves. Especially 16:3 contents were lower—4% versus 17%. Additionally, the relative amount of 18:3 went down by about 7%. On the contrary, the relative amount of 18:2 increased by about 10%, 16:0 by about 4% and 18:1 and 18:0 by about 3% each. To conclude, *in vitro* conditions lowered the unsaturation of acyl-lipids in comparison to *in vivo* conditions.

### 3.2. Lipid Classes in Analysed Leaves of C. sativa from In Vivo and In Vitro Conditions

To examine lipid classes present in the tested leaves, the chloroform extracts were separated by TLC and further analysed on GC. The fatty acid contents in each lipid class were then summed up and, next, the fatty acid contents in each lipid classes were divided by this sum and multiplied by 100. The results were treated as a relative percentage of a given lipid class in the total acyl-lipids present in the analysed tissues.

**Figure 3:**
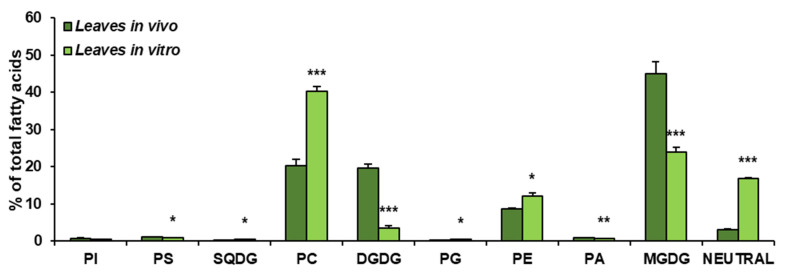
Lipid classes present in *C. sativa* leaves cultured in *in vivo* and *in vitro* conditions. Mean values and SD are presented (data from at least three independent assays). Asterisks indicate significant difference between relative amounts of each lipid class of leaves cultivated *in vivo* and *in vitro* in a two-tailed Student’s *t*-test: * *p* ≤ 0.05, ** *p* ≤ 0.01, *** *p* ≤ 0.001.

The obtained results showed that the growth conditions significantly affected the composition of lipid classes present in leaves of *C. sativa*. *In vivo* leaves contained about 45% of MGDG, about 20% of PC and DGDG (each one), close to 9% of PE, around 3% of neutral lipids (analysed as one class) and small amounts of PI, PS, SQDG, PG and PA—oscillating around 1% for each of them. The dominating lipid class in leaves from *in vitro* conditions was PC, accounting for about 41% of all lipids. MGDG accounted for about 23% and neutral lipids for 18%. PE constituted about 12% of all acyl-lipids and DGDG about 3%. PI, PS, SQGD, PG and PA were present in very small amounts, similarly to leaves from *in vivo* conditions. Thus, *in vitro* conditions significantly diminished the galactolipid contents—from about 65% to about 26% of all acyl-lipids. A significant increase was noted in the case of PC (from 20% *in vivo* to about 41% in *in vitro* conditions) and neutral lipids (analysed as one class)—by about 15%. PE contents increased by about 3% (Figure 3).

### 3.3. Fatty Acid Composition of Main Lipid Classes of C. sativa Leaves from In Vivo and In Vitro Conditions

Detailed analyses of fatty acid compositions were performed for the two main phospholipids (PE and PC) and for the two main glycolipids (MGDG and DGDG). All analysed lipids contained significant amounts of the following fatty acids: 16:0, 18:0, 18:1, 18:2 and 18:3, and small amounts of 20:3, 24:0 and 24:1 (presented in the table as a sum with the name “others”). Galactolipids additionally comprised 16:3 (Table 1). The relative amount of detected fatty acids depended on the analysed lipid and growth conditions.

PE from *in vitro* conditions contained, e.g., 16:0, 18:2 and 18:3 in almost equal amounts (25–28%). Additionally, it contained about 10% of 18:1, 5% of “others” and 4% of 18:0. PE of leaves from *in vivo* condition contained similar amounts of 18:0, 18:2 and 18:3; however, the relative amount of 16:0 increased to about 37% and 18:1 and “others” decreased to 6 and 1.5%, respectively.

The main fatty acid of PC of *C. sativa* leaves was 18:3. However, in PC of leaves from *in vivo* conditions it constituted about 68% of all fatty acids, and in PC of leaves from *in vitro* conditions it constituted only about 46%. On the other hand, *in vitro* conditions strongly increased 18:1 and 18:2 contents (from 0.9 to 9.7% and from 4.3 to 17.7%, respectively). The relative amounts of 16:0, 18:0 and “others” were similar in both growth conditions.

MGDG of *C. sativa* leaves from both growth conditions contained predominantly trienoic fatty acids (18:3 and 16:3). However, the *in vitro* conditions decreased their amount substantially. The relative contents of 16:3 went down from 30% to about 19% and 18:3 from about 63 to about 59%. In contrast, *in vitro* conditions elevated the relative amount of 16:0 (from 4 to 6%), 18:1 (from 0.5 to 3.6%) and 18:2 (from 3 to 11%).

The fatty acid composition of DGDG of *C. sativa* leaves was also affected by the growth conditions. The relative amount of 18:1 accounted for about 12% in *in vivo* conditions and for about 9% in *in vitro* conditions. The contents of 18:3 also decreased from 30% to about 25% and 16:3 from 5.7% to 4.8%. The *in vitro* conditions increased, on the other hand, the relative amount of 16:0 (by about 4%) and 18:2 (by about 5.5%).

### 3.4. Activity and Substrate Specificity of Acyl-CoA:lysophospholipid Acyltransferases of C. sativa Leaves from In Vivo and In Vitro Conditions

**Figure 4:**
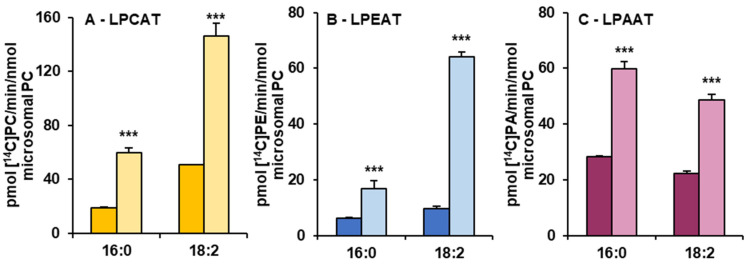
The activity of LPCAT (**A**), LPEAT (**B**) and LPAAT (**C**) enzymes of microsomal fractions of *C. sativa* leaves from two different growth conditions: *in vivo* and *in vitro*. Assays contained appropriate *sn*-1-LPL as fatty acid acceptor and 16:0-CoA or 18:2-CoA (indicated on the figure as 16:0 and 18:0, respectively) as fatty acid donors. Mean values and SD are presented (data from at least three independent assays). Asterisks indicate significant difference between the activity of the tested LPLATs (in assays with tested acyl donors) present in leaves cultivated *in vivo* (left bar) and *in vitro* (right bar) in a two-tailed Student’s *t*-test: *** *p* ≤ 0.001.

Microsomal fractions prepared from leaves of *C. sativa* from *in vivo* and *in vitro* conditions were used in assays evaluating the activity and specificity of acyl-CoA:lysophospholipid acyltransferases. Three types of assays were performed, specific for LPCATs, LPEATs and LPAATs. The results of assays with appropriate lysophospholipids together with 16:0-CoA or 18:2-CoA were selected for the presentation of the activity of the mentioned types of acyltransferases in the analysed microsomal fractions. The activity is shown as pmol of *de novo* synthesised [^14^C]phospholipid during 1 min by aliquots of microsomal fractions containing 1 nmol of microsomal PC (approximately 0.44 µg of microsomal proteins). As a product of LPCAT, LPEAT and LPAAT actions, [^14^C]PC, [^14^C]PE and [^14^C]PA, respectively, were considered. All of the assayed LPLAT activity was significantly higher in microsomal fractions of leaves from *in vitro* conditions. For LPCAT activity it was 2.8 and 2.5 times higher, for LPEAT activity 3.8 and 7 times higher and for LPAAT activity 2 and 2.5 times higher in assays with 16:0-CoA and 18:2-CoA, respectively. Both in assays with microsomal fractions from *in vivo* and *in vitro* conditions, LPCATs and LPEATs utilised 18:2-CoA about 2 to 3.5 times better than 16:0-CoA. On the contrary, LPAATs utilised 16:0-CoA about 1.2 times better than 18:2-CoA (Figure 4).

For the detailed substrate specificity analyses of the tested LPLATs towards acyl-CoAs, assays containing combinations of appropriate lysophospholipids (LPC, LPE and LPA, respectively, for assaying LPCAT, LPEAT and LPAAT specificity) with 10 different acyl-CoAs were conducted. To facilitate the comparison of the affinity of the tested enzymes towards the tested acyl-CoAs, the obtained activities are shown as the percentage of activity towards 16:0-CoA (reference activity).

**Figure 5:**
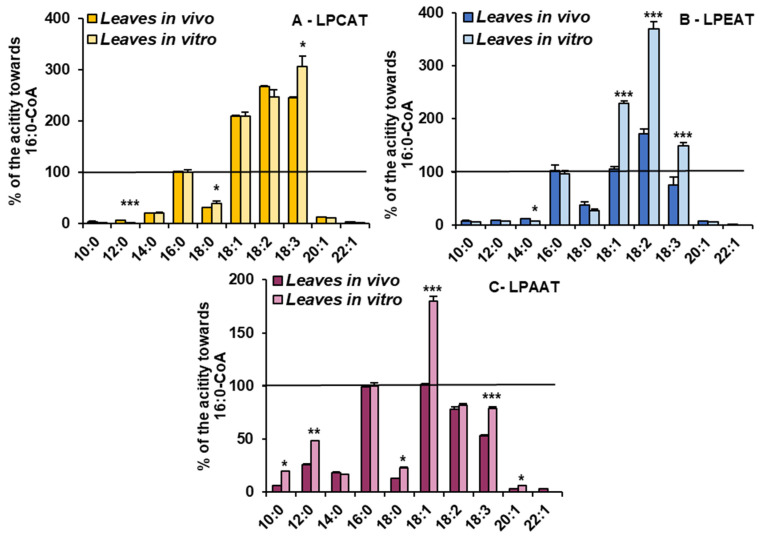
Acyl-CoA (indicated on the figure only by acyl component) preferences of LPCAT (**A**), LPEAT (**B**) and LPAAT (**C**) enzymes present in *Camelina sativa* leaves cultivated in two different growth conditions: *in vivo* and *in vitro*. Mean values and SD are presented (data from at least three independent assays). Asterisks indicate significant difference between the relative activity towards given acyl donor in assays with microsomal fractions of leaves cultivated *in vivo* and *in vitro* in a two-tailed Student’s *t*-test: * *p* ≤ 0.05, ** *p* ≤ 0.01, *** *p* ≤ 0.001.

The LPCATs present in microsomal fractions from both *in vivo* and *in vitro C. sativa* leaves showed the highest activity towards acyl-CoA with 18C unsaturated fatty acids. The activity was 2–3 times higher than towards 16:0-CoA. Growth conditions only slightly modified their specificity. LPCATs from *in vitro* conditions utilised 18:3-CoA the most efficiently and from *in vivo* conditions 18:2-CoA. The relative affinity towards the other tested acyl-CoAs was much smaller than towards 16:0-CoA and was similar in assays with both types of microsomal fractions (Figure 5).

However, the *in vitro* conditions significantly increased the relative activity of LPEATs towards 18:1-CoA (from equal to activity towards 16:0-CoA to about two times higher), 18:2-CoA (from about 150% to about 370%) and towards 18:3-CoA (from 80% to about 150% of activity towards 16:0-CoA). The relative activity of LPEATs towards 10:0-CoA, 12:0-CoA, 14:0-CoA, 20:1-CoA and 22:1-CoA oscillated between 5 and 20% of the reference activity and was a bit lower in assays with *in vitro* microsomal fractions. Additionally, activity towards 18:0-CoA went down from about 37% to about 27% of the reference one (Figure 5).

The relative activity of LPAATs from *in vitro* conditions increased for most of the tested acyl-CoAs compared to LPAATs from *in vivo* conditions. The most striking was the increase in preferences towards 18:1-CoA (from equal to the activity towards 16:0-CoA to 1.8 times higher). The activity towards 10:0-CoA increased from 6% to 20% of the reference activity, towards 12:0-CoA from 18% to 48%, towards 18:0-CoA from 12% to 23% and towards 20:1-CoA from 3% to 6%. The activity towards 18:3-CoA increased from about 53% to 79% of the reference activity and towards 14:0-CoA and 18:2-CoA remained similar, like in assays with microsomal fractions from *in vivo* conditions (Figure 5).

### 3.5. Phospholipid Remodelling Intensity in C. sativa Leaves from In Vivo and In Vitro Conditions

Assays evaluating phospholipid remodelling intensity were performed with microsomal fractions from *C. sativa* leaves from *in vivo* and *in vitro* conditions. Each assay contained only [^14^C]acyl-CoA and aliquots of the tested microsomes. Assays were incubated for 5 and 60 min and the radioactivity in PC, PE and PA of the obtained chloroform extracts was measured. From the amount of *de novo* synthesised appropriate [^14^C]-phospholipids during 60 min incubation, we subtracted those lipids that were synthesised during the 5 min incubation period. We expected that during that time all the endogenous lysophospholipids should have been totally used, as forward reaction performed by LPLAT enzymes is very fast [19]. Thus, the remaining *de novo* synthesised [^14^C]-phospholipids were treated as the result of the remodelling process.

The remodelling intensity of PC, PE and PA evaluated in this way was from about 3 to 4 times faster in assays with a microsomal fraction of *in vitro* leaves than in assays with a microsomal fraction of *in vivo* leaves (Table 2). The acyl-CoA present in assays also affected the remodelling intensity. The highest remodelling intensity was in assays with [^14^C]18:1-CoA followed by assays with [^14^C]18:2-CoA and [^14^C]18:3-CoA. In the case of PA remodelling intensity, the differences between assays with the last two mentioned acyl-CoAs were not as clear. The intensity of remodelling in assays with 18:1-CoA was about 1.7–2.8 times higher than in assays with the two other acyl-CoAs.

Assuming that the proportions between PC, PE and PA in microsomal fractions were similar to those in leaves used for their preparation (see Figure 3), the complete turnover of fatty acids (in assays with 18:1-CoA) of PC should take about 10 h in *in vitro* conditions and about 39 h in *in vivo* conditions. In the case of PE, it would be about 17 h and 95 h, respectively, and in the case of PA about 2 h and 6 hrs. As the deacylation (part of the acyl edition process) of phospholipids undergoing remodelling is faster in the *sn*-2 position than at the *sn*-1 position, the time of turnover of fatty acids at the *sn*-2 position should be shorter and at the *sn*-1 position should be longer than presented above (calculated for all fatty acids present in both *sn*-1 and *sn*-2 positions) [23].

## 4. Discussion

*In vitro* methods of plant cultivation are currently widely used both in agricultural practice and in scientific research. The results obtained from plants from *in vitro* conditions are often extrapolated to *in vivo* conditions. However, the environment of plants growing *in vitro* is completely different from that *in vivo*. Thus, the metabolic processes occurring *in vitro* can also deviate from those from *in vivo* conditions. Despite this, research concerning such effects is rather rare, especially in the area of plant lipid biochemistry. To bridge this gap in knowledge, in the presented studies we investigated the effects of *in vitro* conditions on lipid contents and composition, on the activity and substrate specificity of LPLAT enzymes (the most important enzymes in the acyl editing process of phospholipids) as well as on the intensity of the phospholipid remodelling of *C. sativa* leaves.

### 4.1. In Vitro Growth Conditions Clearly Decline Prokaryotic Pathway Activity

So far, most of the research concerning acyl-lipid contents and composition has been conducted on *A. thaliana*. Data on *C. sativa* are still missing. Our results concerning the fatty acid composition of acyl-lipids in *C. sativa* leaves show that this plant possesses a similar composition of fatty acids to *A. thaliana*, both when plants are cultured in *in vivo* [16,34,35] and in *in vitro* conditions [31]. The composition of individual lipid classes in leaves from *in vivo* conditions also seems to be comparable to that of *A. thaliana* [16]. However, none of these studies concerns comparative analysis between the two types of plant cultivation conditions.

Significant differences have been noticed for the fatty acid composition of individual lipid classes, especially for PE and PC, which were characterised by the dominance of linolenic acid (18:3) over linoleic acid (18:2) in *C. sativa*, whereas for *A. thaliana* the opposite was observed [34]. In the case of galactolipids, MGDG exhibited similar patterns, while DGDG composition was completely different to that of *A. thaliana*. In DGDG of Arabidopsis leaves, 16:0 significantly dominated over other fatty acids, and in DGDG of *C. sativa* leaves the level of palmitic acid was only a bit higher than the level of 18C unsaturated fatty acids [34]. Hitherto, for *in vitro* conditions such experiments have not been conducted.

One of the most visible discrepancies in the fatty acid composition of acyl-lipids of *C. sativa* leaves from *in vivo* and *in vitro* conditions is the amount of trienoic fatty acids. Their relative amount was reduced from about 72% in *in vivo* conditions to about 52% in *in vitro* conditions. With regard to individual lipid classes, a reduction in the relative amount of 18:3 was visible mostly in PC and to some extent in both galactolipids. The reduction in the relative amount of 16:3 primarily concerned the MGDG pool.

Trienoic fatty acids play an essential role in plant adaptation to unfavourable environmental conditions, especially to temperature changes. Their amount rises in cold temperatures and decreases in high temperatures. In addition to boosting tolerance to temperature, these fatty acids are also a source of carbon when they are stored in the TAG pool, are precursors of bioactive molecules and are necessary in stress signalisation [36,37,38,39].

A reduction in trienoic fatty acids has a key effect on membrane fluidity by leading to membrane rigidisation and changes in its conformation. Higashi et al. [40] observed that during stress conditions (heat stress), 18:3 is efficiently removed from the MGDG pool with a simultaneous increase in its level in storage lipids. Similarly to this result, in *C. sativa* leaves cultivated in *in vitro* conditions, we observed an elevated level of neutral lipids with contents of 18:3 increased up to 10% (Supplemental Table S1). Thus, *in vitro* conditions caused a similar effect to the abovementioned heat stress conditions. *In vitro* conditions also caused a significant decline in the galactolipid levels and an increase in phospholipid levels, especially PC and PE. However, as the total acyl-lipid level was about 2.5 times higher in *in vivo* conditions, the absolute amount of PC and PE calculated per unit of dry weight still remained a bit lower than in *in vivo* plants. Thus, *in vitro* conditions visibly decreased prokaryotic pathway activity. The physiological role of this rearrangement in acyl-lipid biosynthetic pathways needs to be elucidated in further studies.

### 4.2. LPLAT Enzyme Activity and Remodelling Intensity Are Enhanced in In Vitro Cultivated Leaves

In the presented studies, we investigated the effect of *in vitro* conditions not only on LPCAT, LPEAT and LPAAT activity, but also on their specificity towards acyl-CoAs. The overall activity of the tested LPLATs was at least 2 times higher in *in vitro* conditions compared to *in vivo*. This could be caused by (i) an increased expression of appropriate genes (causing an increased synthesis of the tested acyltransferases), or (ii) by enhancing posttranslational activity, leading to the increased synthesis of the tested LPLATs, or (iii) by the lower catabolism of these enzymes. According to Klińska et al. [23], the expression level of LPEAT-encoding genes in *C. sativa* leaves from *in vivo* conditions was higher than in their *in vitro* counterparts. Thus, at least in the case of LPEAT enzymes, their higher activity in *in vitro* leaves seems not to be connected with the elevated expression levels of their encoding genes. The physiological significance of enhancing the activity of LPLAT enzymes in *in vitro* conditions might be connected with the observed increased intensity (at least 3 times) of the remodelling of PC, PE and PA. The LPLAT type of enzymes could be involved not only in the synthesis of appropriate phospholipids from lysophospholipids created during the first step of the remodelling process, but also in the creation of the lysophospholipid pool via backward reactions performed by these enzymes [19,20,22]. However, the relative share of the LPLAT type of enzymes in the deacylation process of the mentioned phospholipids needs to be elucidated in further studies as well as the significance of the elevated remodelling intensity of phospholipids for plant development in *in vitro* conditions.

The specificity of LPCATs of *C. sativa* leaves towards acyl-CoA does not differ much from LPCATs from other plants [19,21,41]. All of them were highly specific toward 18C unsaturated fatty acids. *In vitro* conditions also caused only small changes to their preferences to acyl-CoAs. Some differences were noted mostly towards 18:2-CoA and 18:3-CoA. The former favoured acyl-CoA in *in vivo* and the latter in *in vitro* growth conditions.

So far, only the substrate specificity of LPEAT enzymes from *A. thaliana* and *C. sativa* was tested [22,23,42,43]. These studies revealed high preferences of these enzymes towards 16:0-CoA as well as towards unsaturated 18C-acyl-CoAs. We also observed similar preferences in our studies. However, we also noticed that *in vitro* conditions significantly enhanced the affinity of LPEATs towards 18C-unsaturated acyl-CoA compared with the activity towards 16:0-CoA. This could be explained by an enhancement of the expression levels of specific isoforms of LPEATs or by changes in the lipid composition of membranes surrounding the LPEATs. In *C. sativa*, there are six different isoforms of LPEATs and their expression patterns vary in different organs [23]. It has also been shown that isoforms of LPEATs of *C. sativa* present in microsomal fractions of transgenic yeast (harbouring genes encoding these isoforms) expressed affinity towards other acyl-CoAs than LPEATs present in microsomal membranes prepared from *C. sativa* plants [23]. Thus, from the obtained results, we cannot conclude which of these factors is responsible for the observed changes in LPEAT specificity towards acyl-CoA in *in vitro* conditions.

The substrate specificity of LPAAT enzymes was tested in many plant species, such as *Brassica napus, Camelina sativa, Limananthes alba, Linum usitatissimum, Ricinus communis, Syagrus cocoides Martius* and *Zea mays* [22,44,45,46,47]. All of the mentioned studies revealed the diverse substrate specificity of these enzymes, ranging from preference towards short fatty acids to preference towards very-long-chain fatty acids such as 22:1. In our studies, the tested LPAATs of *C. sativa* leaves accepted a wide variety of acyl-CoAs, with the highest preference equally towards 16:0-CoA and 18:1-CoA in *in vivo* conditions. This substrate specificity was to some extent modified by *in vitro* conditions. Generally, most of the acyl-CoAs were better utilised when comparing their activity with the activity towards 16:0-CoA. This could be (similarly to the LPEAT enzymes discussed above) caused by different expressions of specific isoforms of LPAATs in *in vitro* conditions as well as by different environments of LPAAT enzymes. In *C. sativa*, 15 different isoforms encoding LPAATs are present, also exhibiting different expression patterns [48,49]. We have also shown that the membrane lipid compositions of *C. sativa* leaves from *in vivo* and *in vitro* conditions are quite different, which might be an effect of the observed discrepancies.

An interesting finding of our studies is the effect of acyl-CoA on lipid remodelling intensity. The addition of 18:1-CoA to the reaction mixture caused higher acyl editing intensity compared with the addition of 18:2-CoA and especially 18:3-CoA. Similar observations were noticed previously in assays with microsomal fractions of *C. sativa* seeds [21,22] and leaves [23]. Thus, this seems to be a commonplace phenomenon. However, the question remains open concerning the nature of this phenomenon. Are LPLAT enzymes characterised by different deacylation activities after binding different acyl-CoAs? Or are there other deacylation enzymes such as phospholipases or enzymes of the PDAT type that are more active? These questions require further and more detailed studies.

## 5. Conclusions

The presented results clearly indicate that growth conditions significantly influence acyl-lipid metabolism. The discrepancies between *in vivo* and *in vitro* conditions occur in total acyl-lipid contents, in the composition of acyl-lipid classes, in LPLAT enzyme activity and substrate specificity and in phospholipid remodelling intensity. Consequently, the results from experiments conducted on *in vitro* cultivated plants should only be applied to the standard conditions with caution, especially when lipid metabolism is concerned.

## Figures and Tables

**Table 1:** Distribution of different fatty acids in major/chosen phospholipids and galactolipids present in *C. sativa* leaves cultured in *in vivo* and *in vitro* conditions. In “others” pool, 20:1, 20:2, 20:3, 22:0, 24:0 and 24:1 are present. Mean values and SD are presented (data from at least three independent assays). Asterisks indicate significant difference between relative amounts of fatty acids present in each analysed lipid class in leaves cultivated *in vivo* and *in vitro* in a two-tailed Student’s *t*-test: * *p* ≤ 0.05, ** *p* ≤ 0.01, *** *p* ≤ 0.001.

Lipid Class	Type of Cultivation Condition	Fatty Acids (mol%)						
		16:0	16:3	18:0	18:1	18:2	18:3	Others
PE	*In vitro*	25.5	-	4.0	10.6 ***	26.4	28.4	5.0 **
		±0.2		±0.01	±0.1	±0.2	±0.8	±0.2
	*In vivo*	37.4	-	4.8	5.9	24.0	26.3	1.5
		±3.0		±0.4	±0.3	±1.8	±1.7	±0.01
PC	*In vitro*	20.8	-	4.2	9.7 ***	17.7 ***	46.5 ***	1.2
		±0.3		±0.01	±0.1	±0.1	±0.2	±0.1
	*In vivo*	21.9	-	3.2	0.9	4.3	68.0	1.4
		±2.5		±1.01	±0.1	±0.2	±3.0	±0.2
MGDG	*In vitro*	6.2 **	19.4 **	-	3.6 ***	11.4 ***	58.8 **	0.6
		±0.1	±0.3		±0.1	±1.3	±0.9	±0.01
	*In vivo*	4.0	30.0	-	0.5	3.0	62.6	-
		±0.2	±1.4		±0.4	±0.4	±0.6	
DGDG	*In vitro*	37.4	4.8	-	8.6*	23.4 *	25.4 **	0.5
		±1.3	±0.1		±0.6	±0.6	±0.1	±0.01
	*In vivo*	33.4	5.7	-	12.2	17.9	30.8	-
		±1.0	±0.3		±1.0	±0.8	±0.8	

**Table 2:** Remodelling intensity—[^14^C]acyl group incorporation from [^14^C]acyl-CoA into PC, PE and PA of microsomal fractions of *Camelina sativa* leaves cultivated in *in vivo* and *in vitro* growth conditions. Asterisks indicate significant difference between remodelling intensity (in assays with each tested acyl donor) of indicated phospholipids of microsomal fractions from leaves cultivated *in vivo* and *in vitro* in a two-tailed Student’s *t*-test, ** *p* ≤ 0.01, *** *p* ≤ 0.001.

Type of Cultivation Condition	Fatty Acid Donors	Remodelling Intensity (pmol [^14^C]PL/nmol Microsomal PC/min)		
		PC	PE	PA
*In vitro*	[^14^C]18:1-CoA	3.18 *** ±0.07	0.59 *** ±0.01	0.26 *** ±0.02
	[^14^C]18:2-CoA	1.56 *** ±0.05	0.31 *** ±0.02	0.1 ** ±0.008
	[^14^C18:3-CoA	1.3 *** ±0.09	0.21 *** ±0.007	0.12 *** ±0.009
*In vivo*	[^14^C]18:1-CoA	0.85 ±0.01	0.15 ±0.01	0.03 ±0.002
	[^14^C]18:2-CoA	0.47 ±0.01	0.09 ±0.005	0.03 ±0.001
	[^14^C18:3-CoA	0.43 ±0.02	0.07 ±0.005	0.06 ±0.001

## Data Availability

The data presented in this study are available on request from the corresponding author. The data are not publicly available due to privacy.

## Supplementary Materials

The following are available online at https://www.mdpi.com/article/10.3390/cells10092326/s1, Table S1: Relative amount of different fatty acids in neutral lipid classes present in *C. sativa* leaves cultured in *in vivo* and *in vitro* conditions.
