# Supplementary material for: In Vitro Growth Conditions Boost Plant Lipid Remodelling and Influence Their Composition
Source: Cells. 2021 Sep 6;10(9):2326. doi: 10.3390/cells10092326 (PMC8472737; doi:10.3390/cells10092326)
Supplement: Supplementary file 1 [file cells-10-02326-s001.zip › cells-1363794-supplementary.pdf]

Cells 2021

In vitro Growth Conditions Boost Plant Lipids Remodelling and Influence Their Composition

Sylwia Klińska, Sara Kędzierska, Katarzyna Jasieniecka-Gazarkiewicz, Antoni Banaś

Supplemental

Supplemental Table S1. Relative amount of different fatty acids in neutral lipid classes present in *C. sativa* leaves cultured in *in vivo* and *in vitro* conditions. In 'other' pool are present: 20:0, 20:1, 20:2, 20:3, 22:0, 22:1, 24:0, 24:1. Mean values and SD are presented (data from at least three independent assays). Asterisks indicate significant difference between relative amounts of given fatty acid in analysed lipid class of leaves cultivated *in vivo* and *in vitro* in a two-tailed Student's t-test: * $p \leq 0.05$, ** $p \leq 0.01$, *** $p \leq 0.001$.

Lipid class	Type of cultivation condition	Fatty acids [mol%]						
		**16:0**	**16:3**	**18:0**	**18:1**	**18:2**	**18:3**	**other**
**Neutral lipids**	*in vivo*	31.8** ± 1.5	0.4 ± 0.01	14.8** ± 0.1	5.3** ± 0.3	10.4* ± 0.07	21.9** ± 1.9	15.5** ± 1.0
	in vitro	23.2 ± 1.4	0.16 ± 0.01	8.2 ± 0.2	10.4 ± 0.2	16.9 ± 1.1	31.3 ± 1.8	9.9 ± 2.1
